# Single-molecule dynamic DNA junctions for engineering robust molecular switches†
†Electronic supplementary information (ESI) available: Experimental section, supplementary tables and figures. See DOI: 10.1039/c9sc03389k


**DOI:** 10.1039/c9sc03389k

**Published:** 2019-10-07

**Authors:** Shuang Cai, Yingnan Deng, Shengnan Fu, Junjie Li, Changyuan Yu, Xin Su

**Affiliations:** a College of Life Science and Technology , Beijing University of Chemical Technology , Beijing 100029 , China . Email: xinsu@mail.buct.edu.cn

## Abstract

DNA molecular switches have emerged as a versatile and highly programmable toolbox and are extensively used in sensing, diagnosis, and therapeutics. Toehold mediated strand displacement serves as the core reaction for signal transduction and amplification. However, the severe leakage of this reaction limits the development of scalable and robust circuits. We engineered single-molecule dynamic DNA junctions for developing ‘zero-leakage’ molecular switches which are responsive to various inputs. Input binding enhances dynamic junctions' stability allowing for the transient binding of fluorescent probes as the output signal. Unlike the conventional intensity-based output, this molecular switch provides remarkably distinguishable kinetics-based outputs permitting ruling out leakage signals at the single-molecule level. The inputs are detected with significant sensitivity without using any amplification step. It is also revealed that the output signal is sensitive to the binding affinity of inputs and their recognition elements making the molecular switch a potential affinity meter. Considering these features, we anticipate that it would find broad applications in large-scale DNA circuits, responsive materials, and biomolecule interaction study.

## Abstract 2

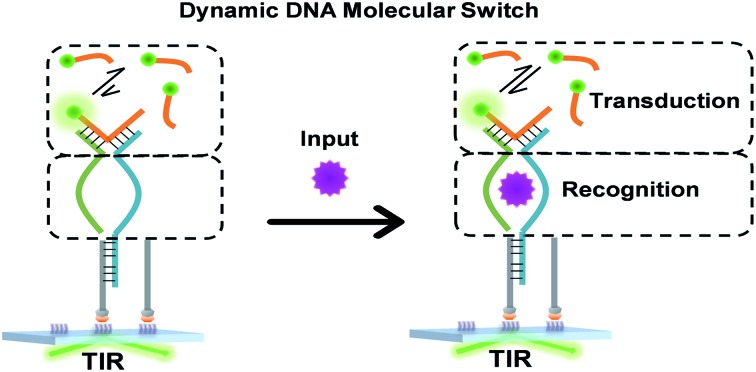
Highly robust DNA molecule switches were engineered by utilizing single-molecule dynamic three-way junctions.

## Introduction

Owing to the programmable language of base pairing, DNA nanotechnology has drawn widespread attention for engineering a variety of functional molecular devices which are extensively used in sensing,^1^ diagnosis,^2^ and therapeutics.^3^ Dynamic DNA nanostructures incorporating functional nucleic acids such as aptamers and DNAzymes that are responsive to chemical/biochemical stimuli have been utilized to develop smart responsive systems.^4^ DNA molecular switches are a class of rationally designed dynamic nanostructures that utilize a programmable network of reactions to execute complex algorithms in order to process molecular information and computation.^5^,^6^ DNA molecular switches translate the language of living organisms and have helped in developing micro- and nanoscale tools for sensing, adapting and making decisions. Such molecular switches are therefore suited for numerous applications in nanotechnology, and increasing efforts are being directed toward their engineering.

In a typical DNA molecular switch, recognition and signal transduction elements are necessary to provide affinity and specificity. Amplification steps are always exploited to enhance signals. Toehold mediated strand displacement (TMSD) has emerged as a unique tool for signal transduction, in which inputs induce structure switching to activate the invading strand of TMSD.^7^–^10^ Strand displacement-based amplification strategies are also frequently adopted for molecular switches.^11^–^13^ However, the greatest challenge for a TMSD-based molecular switch is the initiation of the reaction in the absence of an input, known as leakage, contributing to a non-negligible amount of background, which limits the engineering of more specific and sensitive tools.^14^–^16^ Efforts have been made to reduce the leakage, such as scalable junction configurations,^17^ toeless strategies,^16^ and mismatch involved TMSD.^18^ It is still desirable to develop a new strategy to reduce leakage and guarantee high sensitivity for engineering highly robust DNA molecular switches.

Single-molecule analysis represents a promising advance in the field of DNA nanotechnology.^19^,^20^ Observing the dynamics in nanostructures at the single-molecule level can be helpful to develop more robust molecular switches or circuits. Transient binding between short oligonucleotides and their complementary sequences creates featured single-molecule kinetics on total internal reflection fluorescence microscopy (TIRF) to permit a super-resolution imaging technique called DNA-PAINT.^21^–^23^ Transient binding mode is not only an essential component for super-resolution imaging but also useful for highly sensitive and specific target detection.^24^,^25^ Due to the distinct single-molecule kinetics, targets can be arbitrarily distinguished from the background. We developed a series of analytical tools based on single-molecule DNA transient binding; however, the simple two-strand mode is not suitable for scalable and complex molecular switches or circuits.^26^–^28^

Here, we engineered single-molecule dynamic DNA junctions for constructing robust molecular switches which are responsive to various inputs with high sensitivity and in a ‘zero-leakage’ manner. The basic structure of the DNA molecular switches is a dynamic three-way junction (TWJ) that consists of a recognition domain and transduction domain. Input binding induces the stability alteration of the TWJ allowing the transient binding of fluorescent probes which is monitored by TIRF. Single-molecule kinetics as the molecular switch output allows for identifying inputs with high-confidence at the single-molecule level. The inputs are detected with significant detection limits for nucleic acid (10 fM), protein (20 pM), and small molecules (50 pM). The output signal of the molecular switch also serves as a binding affinity meter measuring the interaction of ligands and their receptors. With the above-mentioned merits, it would find broad applications in large-scale DNA circuits, responsive materials, and biomolecule interaction studies.

## Results and discussion

### Design of the TWJ-based DNA molecular switch

**Scheme 1:**
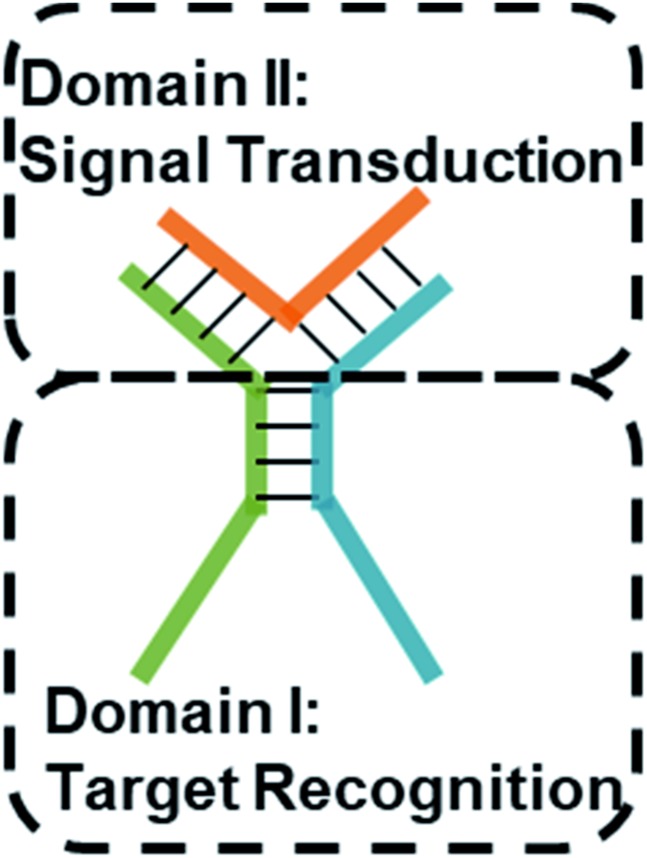
Structure illustration of a TWJ-based DNA molecular switch. Domains I and II are responsible for target recognition and signal transduction, respectively. Domain I can be functional nucleic acids such as aptamers.

Multi-way junctions are a widely used structure for constructing DNA-based devices.^15^,^29^ Generally, the highlighted two domains I and II can serve as target recognition and signal transduction, respectively. Target binding on domain I can alter the stability of domain II facilitating downstream reactions (*e.g.* TMSD and DNAzyme catalysis) resulting in signal transduction (Scheme 1). We engineered dynamic TWJs with 10–14 bp in domain II (*e.g.* FP1, B1, and G1, for sequences see Table S1 ESI†). Target binding would induce a significant change in the TWJs' stability which can be characterized by single-molecule kinetics analysis (Scheme 1).

### Single-molecule characterization of the dynamic TWJs

**Fig. 1:**
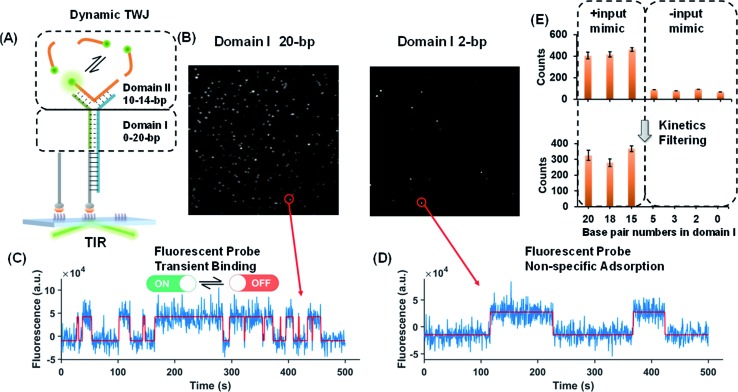
(A) Schematic illustration of the dynamic TWJ. The dashed lines represent the various numbers of base pairs. (B) Single-molecule fluorescence images of the TWJs (12 bp in domain II): left, 20 bp in domain I; right, 2 bp in domain I. (C) Representative single-molecule fluorescence–time trajectory that reflects the multiple binding and dissociation of the fluorescence probe in the TWJ. (D) Single-molecule fluorescence–time trajectory of the nonspecific surface adsorption of the fluorescence probe. (E) Single-molecule counts with/without kinetics filtering (to remove the nonspecific signals) as a function of the numbers of base pairs in domain I.

Dynamic TWJs with different configurations were first exploited to mimic the input recognition of the DNA molecular switch. As shown in Fig. 1A, TWJs with long and short associate (base-paired) regions in domain I were designed to mimic the input-binding DNA switch and the off-input DNA switch, respectively. The short domain II in the TWJs allows for the transient binding of the fluorescent probe to form a binding-dissociation equilibrium. The TWJs were immobilized on the imaging surface using the biotinylated capture probe (Fig. 1A). The formation of the DNA structures was confirmed using native PAGE gel (Fig. S1, ESI†). In TIRF, only fluorescent probes entering into the evanescent field (∼100 nm above the surface) are illuminated, and only when they bind to the domain II sequence will they stay in place long enough for a signal to be detected using a camera integration time of 500 ms. The fluorescence of the single TWJs was detected using the camera, and the TWJs with a long associate region in domain I give many observed single molecules (Fig. 1B); moreover, many emitter alterations between ON and OFF states in the single-molecule fluorescence trajectories were observed (Fig. 1C). In contrast, much fewer single molecules and alterations were found when the TWJs have a short associate region in domain I (Fig. 1D). The emitter alteration represents the flux of fluorescent probes. The trajectories with many alterations originate from the transient binding of the fluorescent probe in the domain II, whereas the trajectories with few alterations can be attributed to the non-specific adsorption of the fluorescent probes (leakage). These results suggest that the short associate region of domain I is not sufficiently stable to provide accessibility for the fluorescent probes. By applying kinetics filters (see the Experimental section, ESI†), the TWJs can be identified at the single-molecule level with high confidence and the nonspecific binding is ruled out (Fig. 1E). The TWJ can be designed flexibly such that high S/N can be achieved in a variety of structures (Fig. 1E and S2, ESI†). Sophisticated optimization is not needed. In addition, this system exhibits high tolerance to salinity (Fig. S3, ESI†). We speculated that the single-molecule dynamic TWJ would allow for ‘zero-leakage’ computation.

### Nucleic acids as the input of the TWJ-based DNA molecular switch

**Fig. 2:**
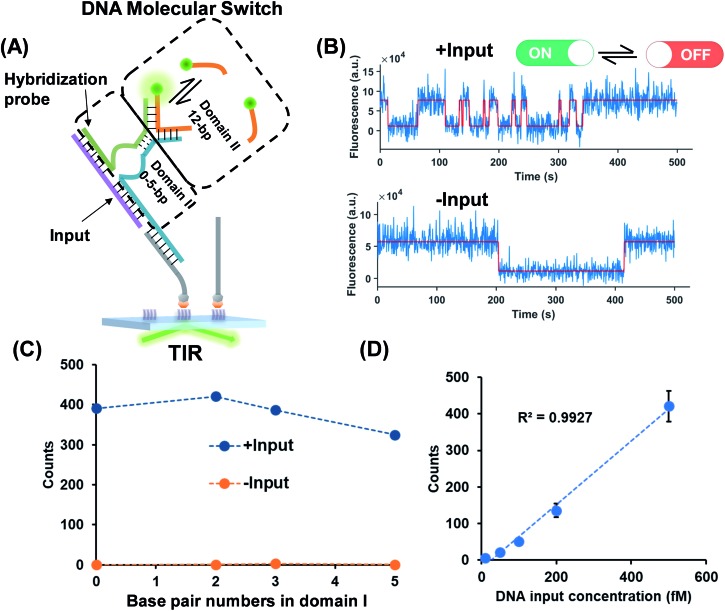
(A) ssDNA as the input of the molecular switch in which the hybridization probe was designed for molecule recognition. The dashed lines represent the various numbers of base pairs in domain I. The base pair number in domain II is 12. (B) Representative single-molecule fluorescence–time trajectories of the molecular switch (top panel, in the presence of inputs; bottom panel, in the absence of inputs). (C) Signal (blue circles) to background (yellow circles) against the numbers of base pairs in domain I. (D) Linear relationship (*R*^2^ > 0.99) of the input concentration and output single-molecule counts. The detection limit for the input is 10 fM calculated as three standard deviations above the blank.

As shown in Fig. 2A, a hybridization probe is attached to the TWJs to recognize nucleic acids. As expected, the single-stranded DNA (ssDNA) input induces the transient binding of fluorescent probes (Fig. 2B). In contrast, only the nonspecific binding of fluorescent probes was found in the absence of inputs because their binding sites are not available (Fig. 2B). The gel characterization of the DNA structures reveals that the presence of inputs results in more bound fluorescent probes, which is consistent with the single-molecule assays (Fig. S4, ESI†). Four molecular switch designs were considered, each varying in terms of the numbers of base pairs in domain I and all of the designs provide high S/N (Fig. 2C). We choose the DNA switch with 2 bp of the associate region in domain I for the quantification of the ssDNA input. The single-molecule counts after kinetics filtering show a linear relationship with the input concentration yielding a detection limit of 10 fM (Fig. 2D). Importantly, ‘zero leakage’ sensing was achieved as the single molecules rising from probes' non-specific binding were ruled out by kinetics filtering.

### Small molecules and protein as inputs of the TWJ based DNA molecular switch

**Fig. 3:**
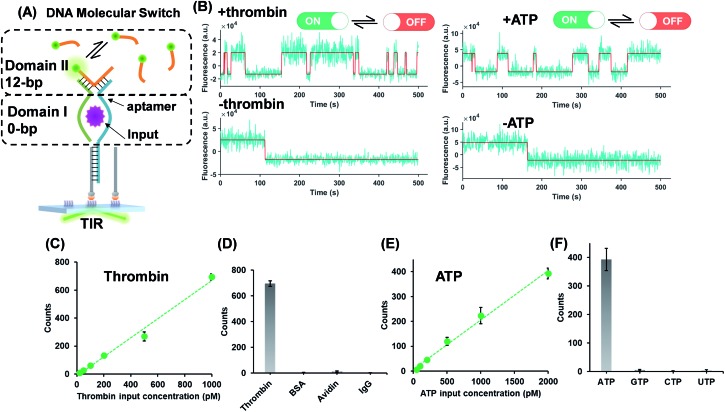
(A) Protein (thrombin) or small molecule (ATP) as the input of the molecular switches in which split aptamers were used as recognition elements. (B) Representative single-molecule fluorescence–time trajectories of the molecular switch (top panel, in the presence of inputs; bottom panel, in the absence of inputs). Linear relations (*R*^2^ > 0.98) of the thrombin (C) or ATP (E) concentration and single-molecule counts of the molecular switch, yielding LODs of 20 pM and 50 pM for thrombin and ATP, respectively. The specificity of the molecular switches for thrombin (D) and ATP (F).

The successful design of the molecular switch for ssDNA highlights the potential for engineering the DNA switch to be a general framework for sensing other biomolecular interactions with known target recognition aptamer sequences without the need to re-design the entire sequences. We choose ATP and thrombin as a representative small molecule and protein, respectively. Split aptamer-based strategies are developed to adopt the ‘sandwich like’ molecule recognition mode of TWJs. The split aptamer fragments can be drawn close to each other only when they bind to a target. The TWJ based molecular switches were engineered with the split aptamers for ATP and thrombin (Fig. 3A). In particular, the thrombin binding aptamers I1 and I2 consist of two G-quartet conformations that selectively bind to specific and different epitopes of thrombin. I1 has a 29 nt DNA aptamer which binds exosite I of thrombin (fibrinogen binding site) with nanomolar affinity, while I2 has a 15 nt DNA aptamer binding to exosite II of thrombin (heparin binding domain) with subnanomolar affinity.^30^,^31^ We chose split aptamer segments (14 nt and 15 nt) derived from a 27 nt sequence (*K*_d_ ∼ 6 μM).^32^,^33^ As expected, the dynamic TWJs allow for the observable transient binding of fluorescent probes in the presence of the inputs. Arbitrary discriminations were achieved as only nonspecific binding was detected in the absence of inputs (Fig. 3B). Standard curves were constructed with the linear portion to quantify the inputs, yielding a LOD of 20 pM and 50 pM for thrombin and ATP, respectively, calculated as three standard deviations above the blank (Fig. 3C and E). High specificity was also achieved, as shown in Fig. 3D and F; no other molecular analogs give significantly higher signals than the blank.

### Molecular switch is sensitive to the binding affinity of the inputs

**Fig. 4:**
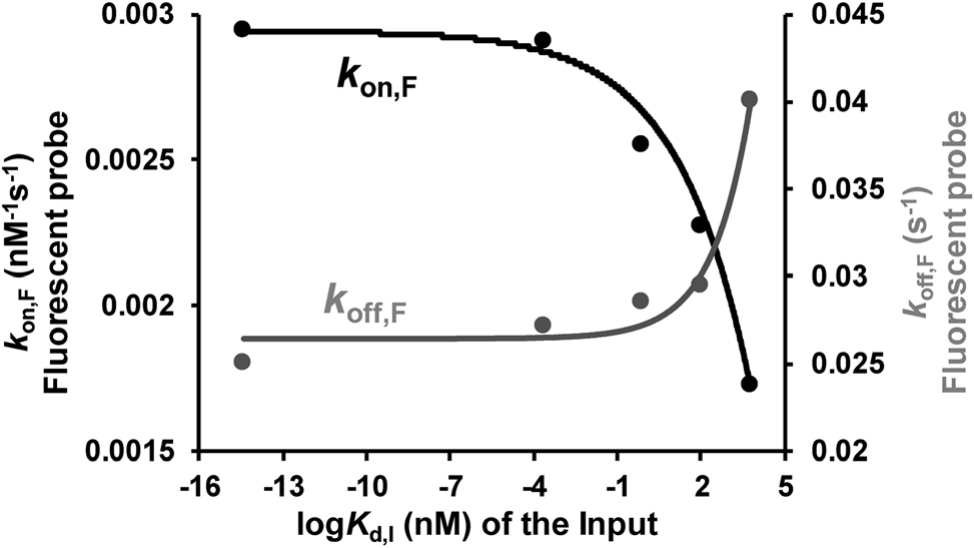
Relationship between the dissociation constant of the inputs (three DNA inputs, ATP, and thrombin) and their recognition elements (*K*_d_,_I_) and the rate constants of the fluorescent probe binding (*k*_on,F_) and dissociation (*k*_off,F_). Kinetics parameters (*k*_on,F_ and *k*_off,F_) can be derived from *τ*_on_ and *τ*_off_ which are shown in Fig. S5, ESI.† *k*_on,F_ and *k*_off,F_ were fit by exponential decay and growth, *k*_on,F_*y* = –4 × 10^–4^ × exp(*x*/3.2) + 3 × 10^–3^ and *k*_off,F_*y* = 8 × 10^–4^ × exp(*x*/1.4) + 0.03, respectively.

Binding affinity is a crucial parameter for biomolecule interaction studies and drug discovery. Binding affinity is typically measured and reported using the equilibrium dissociation constant (*K*_d_), which is used to evaluate and rank order strengths of bimolecular interactions. Molecule probes are exploited to measure *K*_d_. We anticipated that TWJ-based molecular switches may be used for measuring the *K*_d_ of inputs because the binding affinity may affect the single-molecule kinetics of the fluorescent probes. The transient binding of fluorescent probes is monitored at the single-molecule level by TIRF, and the dwell times in the fluorescence-ON (*t*_on_) state and fluorescence-OFF (*t*_off_) state are both exponentially distributed (Fig. S5, ESI†).^34^,^35^ Fitting of single-exponential distributions to the experimental dwell time distributions yield the time constants *τ*_on_ and *τ*_off_, respectively. The binding of the fluorescent probe can be approximated as a bimolecular reaction in the input bound state. Accordingly, fluorescent probes' binding and dissociation kinetics parameters (*k*_on,F_ and *k*_off,F_) can be derived from *τ*_on_ and *τ*_off_. The three inputs have different binding affinities where the *K*_d_ of the nucleic acid input is predicted using NUPACK.^36^ As shown in Fig. 4, *k*_on,F_ decreases exponentially with the *K*_d_ of the input, whereas *k*_off,F_ shows the opposite trend. This suggests that the molecular switch is more sensitive to the binding affinity at nanomolar and micromolar levels which are very common in aptamer–ligand^37^ and protein–protein interactions.^38^,^39^ To demonstrate the ability of this molecular switch for measuring binding affinity, we tested the *K*_d_ of 17β-estradiol and its aptamer.^40^ Likewise, the dwell times of the fluorescent probes were measured in the presence of 17β-estradiol (Fig. S5†), and the kinetics parameters were obtained. By using the curves in Fig. 4, the *K*_d_ of 17β-estradiol was determined as 18 nM which is close to the reported value.^41^ The transient binding of nucleic acid is highly sensitive to salinity,^42^ and high or low pH may affect the stability of nucleic acids. Accordingly, the molecular switch is suitable for comparing different interactions under the conditions with certain salinity and neutral pH (*e.g.* evaluating different aptamers for a certain ligand).

## Conclusions

In summary, robust DNA molecular switches based on single-molecule dynamic TWJs were developed which are suited for a variety of inputs. Single-molecule kinetics as the output permits arbitrary discrimination of input signals and leakage signals. No amplification step is used in the molecular switch as the single-molecule assay provides enough sensitivity. It is also revealed that the output is sensitive to the binding affinity of inputs with their recognition elements making the molecular switches a potential affinity meter. Considering these features, single-molecule dynamic DNA junctions have great potential to further explore versatile DNA nanodevices for the realization of more complex, robust, scalable, and intelligent systems.

## Conflicts of interest

There are no conflicts to declare.

## Supplementary Material

Supplementary information
