# Supplementary material for: Single-molecule dynamic DNA junctions for engineering robust molecular switches
Source: Chem Sci. 2019 Oct 7;10(43):9922–7. doi: 10.1039/c9sc03389k (PMC7006622; doi:10.1039/c9sc03389k)
Supplement: Supplementary file 1 [file SC-010-C9SC03389K-s001.pdf]

Electronic Supplementary Material (ESI) for Chemical Science.
This journal is © The Royal Society of Chemistry 2019

Electronic Supplementary Information

Single-Molecule Dynamic DNA Junctions for Engineering Robust Molecular Switches

Shuang Cai,[a,#] Yingnan Deng,[a,#] Shengnan Fu,[a] Junjie Li,[a] Changyuan Yu,[a] and Xin Su[a,*]

[a] College of Life Science and Technology, Beijing University of Chemical Technology, Beijing 100029, China.

[*] Corresponding author

[#] These authors contributed equally.

Email: xinsu@mail.buct.edu.cn

Tel: +86-10-64421335

Fax: +86-10-64416248

Tables of content

Experimental Section.

Sequence of the oligonucleotides used in this work in Table S1.

Supplementary Figures.

Experimental Section

Oligonucleotides and other chemicals. All of the oligonucleotides used in this work were synthesized by Sangon Co. (Shanghai, China), modified oligonucleotides were purified by HPLC, while unmodified oligonucleotides were purified by PAGE. The sequences are listed ESI. Thrombin and ATP were also from Sangon Co. (Shanghai, China). (3-Aminopropyl) triethoxysilane (APTES), 3,4-dihydroxybenzoate (PCA), protocatechuate dioxygenase (PCD), and Trolox were obtained from Sigma-Aldrich (St. Louis, MO). mPEG-succinimidyl valerate (mPEG-SVA, MW, 5000), biotin-PEG-succinimidyl valerate (biotin-PEG-SVA, MW, 5000), and sulfo-disuccinimidyl tartrate (Sulfo-DST) were obtained from SeeBio Co. (Shanghai, China). All chemicals were used as received without additional purification. DNase/RNase-free deionized water from Tiangen Biotech Co. (Beijing, China) was used in all experiments.

TIRFM setup and single-molecule detection. An inverted microscope (Nikon, ECLIPSE, Ti−U) equipped with a TIRF objective (Nikon, 100× magnification, 1.49 NA) was used for single-molecule assays. The fluorophore was excited by a 520 nm laser, and the fluorescence emission was detected with a filter of 593 nm (Semrock, NY) by an EMCCD camera (Andor, iXon 897). The pixel size of this camera matches very well with the magnification offered by the 100× TIRF objective, giving a final resolution of 0.15 μm per pixel. To immobilize the oligonucleotides and passivate the surface, glass slides were coated with a mixture of PEG and biotinylated PEG with a ratio of 10:1 according to the previously reported method.[1] Sample cells on the slide are constructed by fixing a cut 1-cm length of a pipet tip (Eppendorf) to a coverslip using epoxy adhesive. The slide surface was briefly incubated with TE buffer (10 mM Tris-HCl, 1 mM EDTA, pH 8.0) followed by 1 mg ml^{-1} streptavidin for 15 min. Then, excess streptavidin was flushed with TE buffer. Next, 50 pM of the biotinylated capture probe was added to the sample cell for 15 min, and the excess probe was flushed out by 1× PBS for three times. The dynamic TWJs and dynamic molecular switches were prepared by annealing the component strands from 90 °C to 25 °C for 10 min in 1× PBS, and then added into the sample cell with a final concentration of 100 pM. The formation of the DNA structures was confirmed by native PAGE gel (15%). Next, 20 nM of the Cy3 labeled fluorescent probes in 1× PBS containing an oxygen scavenger system[2] (OSS, 2.5 mM PCA, 25 nM PCD, 1 mM Trolox) were added to the sample cell. The transient binding of

the individual fluorescent probes was monitored the EMCCD camera (500 ms, gain 30). For the single-molecule assays, the room temperature was controlled at 25±1 °C.

Extraction and analysis of single-molecule fluorescence kinetics data. The fluorescence intensity-versus-time trace for each candidate region of interest (ROI) identified from the fluctuation map is generated by custom MATLAB code. The single-particle finding threshold is 3. The traces were idealized by a two-state Hidden Markov model (HMM) to generate the intensity and the dwell times of ON- and OFF- states. Kinetics filters were applied as follow for a candidate to be counted as the TWJ with a long domain I or the DNA molecule switch with target binding:

1) Minimum size of intensity jump (OFF- to ON- state) that counts as a binding/unbinding event is 3-fold of standard deviation.

2) Minimum number of intensity transitions is 10.

3) Minimum median of the dwell times for each state is 3 s.

Table S1 Sequence of the oligonucleotides in this work (presented by figures)

Figure	Name	Color	Sequence (5'-3')
1	FP1	Yellow	Cy3-AAACACGGATCCCATA
1	B1	Blue	TGGGATCTCTTGCCATGCGGTGATCGCCGTACTCT ATGAGCCTCTG
1	CP1	Gray	biotin-TTTTTCAGAGGCTCATAGAGTACG
1	G1 (domain I 0-bp)	Green	CCGTGT
1	G2 (domain I 2-bp)	Green	AGCCGTGT
1	G3 (domain I 3-bp)	Green	GAGCCGTGT
1	G4 (domain I 5-bp)	Green	AAGAGCCGTGT
1	G5 (domain I 15-bp)	Green	ACCGCATGGCAAGAGCCGTGT
1	G6 (domain I 18-bp)	Green	ATCACCGCATGGCAAGAGCCGTGT
1	G7 (domain I 20-bp)	Green	CGATCACCGCATGGCAAGAGCCGTGT
2	FP1	Yellow	Cy3-AAACACGGATCCCATA
2	CP1	Gray	biotin-TTTTTCAGAGGCTCATAGAGTACGG
2	I1	Purple	GCGGTGTGATGGAAGTGGGTGGTAGGTGAGATG GATCG
2	B2	Blue	TGGGATGCACTTTTTTTTTTTTTTTCCACTTCCATCA CACCGCCCGTACTCTATGAGCCTCTG
2	G8 (domain I 0-bp)	Green	CGATCCATCTCACCTACCACTTTTTTTTTTTTTTTTTT TCCGTGT
2	G9 (domain I 2-bp)	Green	CGATCCATCTCACCTACCACTTTTTTTTTTTTTTTTA GCCCGTGT
2	G10 (domain I 3-bp)	Green	CGATCCATCTCACCTACCACTTTTTTTTTTTTTTTTTG CCCGTGT
2	G11 (domain I 5-bp)	Green	CGATCCATCTCACCTACCACTTTTTTTTTTTTTTTAGT GCCCGTGT
3	FP1	Yellow	Cy3-AAACACGGATCCCATA
3	CP1	Gray	biotin-TTTTTCAGAGGCTCATAGAGTACGG
3	B3 (for ATP)	Blue	TGGGATTGGAAGGAGGCGTCCGTACTCTATGAGC CTCTG
3	B4 (for Thrombin)	Blue	TGGGATGGTTGGTGTGGTTGGCCGTACTCTATGAG CCTCTG

3	G12 (for ATP)	Green	TATGAGGGGGTCCACCGTGT
3	G13 (for Thrombin)	Green	TCAGTGGGGTTGGACGGGATGGTGCCTGACCGTG T
4	I1	Purple	GCGGTGTGATGGAAGTGGGTGGTAGGTGAGATG GATCG
4	I2	Purple	AAGTGGGTGGTAGG
4	I3	Purple	GTGGGTGGTA
4	B5 (for 17β-estradiol)	Blue	TAGGGTGCGGCTCTGCGCATTCAATTGCTGCGCGC TGAAGCGCGGAAGCCCGTACTCTATGAGCCTCTG
4	G14 (for 17β-estradiol)	Green	GCTTCCAGCTTATTGAATTACACGCAGAGGGTACC GTGT
S1	FP2	Yellow	AAACACGGATCCCATA
S1	CP2	Gray	TTTTTCAGAGGCTCATAGAGTACGG
S1	B1	Blue	TGGGATCTCTTGCCATGCGGTGATCGCCGTACTCT ATGAGCCTCTG
S1	G1 (domain I 2-bp)	Green	AGCCGTGT
S1	G2 (domain I 20-bp)	Green	CGATCACCGCATGGCAAGAGCCGTGT
S2	FP1	Yellow	Cy3-AAACACGGATCCCATA
S2	B1	Blue	TGGGATCTCTTGCCATGCGGTGATCGCCGTACTCT ATGAGCCTCTG
S2	B6	Blue	ATGGGATCTCTTGCCATGCGGTGATCGCCGTACTC TATGAGCCTCTG
S2	B7	Blue	GGGATCTCTTGCCATGCGGTGATCGCCGTACTCTA TGAGCCTCTG
S2	G1	Green	CGATCACCGCATGGCAAGAGCCGTGT
S2	G15	Green	CGATCACCGCATGGCAAGAGCCGTGTT
S2	G16	Green	CGATCACCGCATGGCAAGAGCCGTG
S4	FP2	Yellow	AAACACGGATCCCATA
S4	CP2	Gray	TTTTTCAGAGGCTCATAGAGTACGG
S4	I1	Purple	GCGGTGTGATGGAAGTGGGTGGTAGGTGAGATG GATCG
S4	B2	Blue	TGGGATGCACTTTTTTTTTTTTTTTCCACTTCCATCA CACCGCCCGTACTCTATGAGCCTCTG
S4	G9	Green	CGATCCATCTCACCTACCACTTTTTTTTTTTTTTTA GCCCGTGT

The sequence color is shown in the corresponding figures. Individual strand is identified by its unique name and may be used in different figures. Note that for Figure S2 the sequences compose the TWJs with different domain II, in which the association region of domain I is 20-bp.

Supplementary Figures

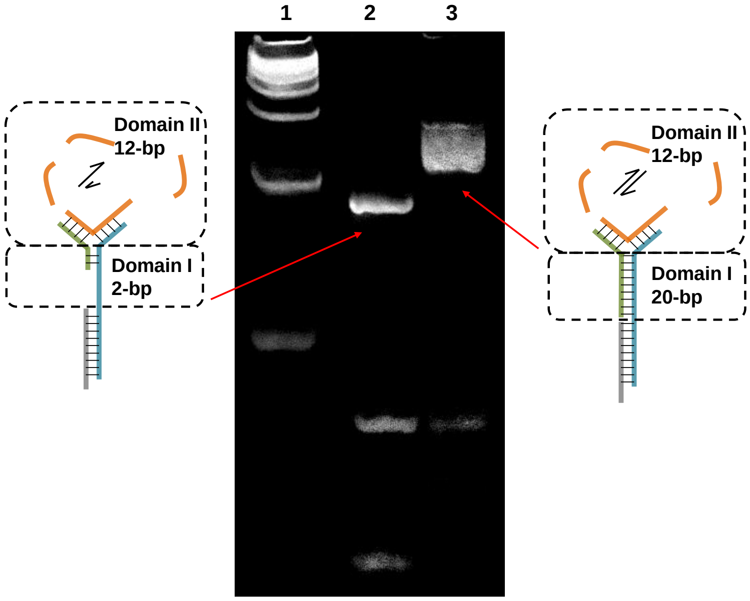

Figure S1 Native PAGE gel characterization of the dynamic TWJs, lane 1: 25-bp marker, lane 2: 2-bp in domain I, lane 3: 20-bp in domain I. The base pair numbers in domain II is 12.

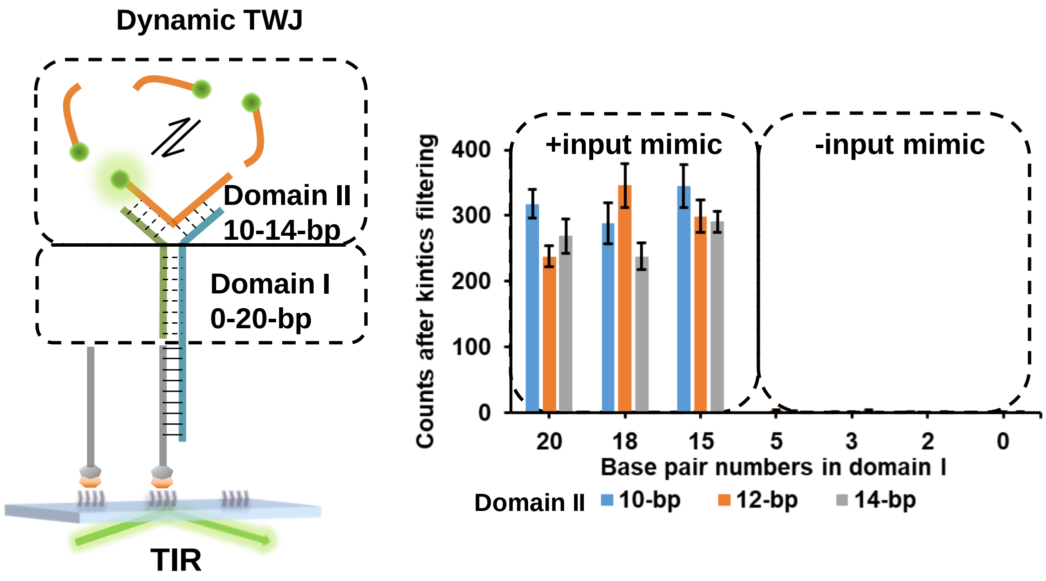

Figure S2 Single-molecule counts of the TWJs after kinetics filtering (to remove the nonspecific signals) as a function of the length of the associate region (base pair numbers) in domain I. The columns represent different structures of domain II.

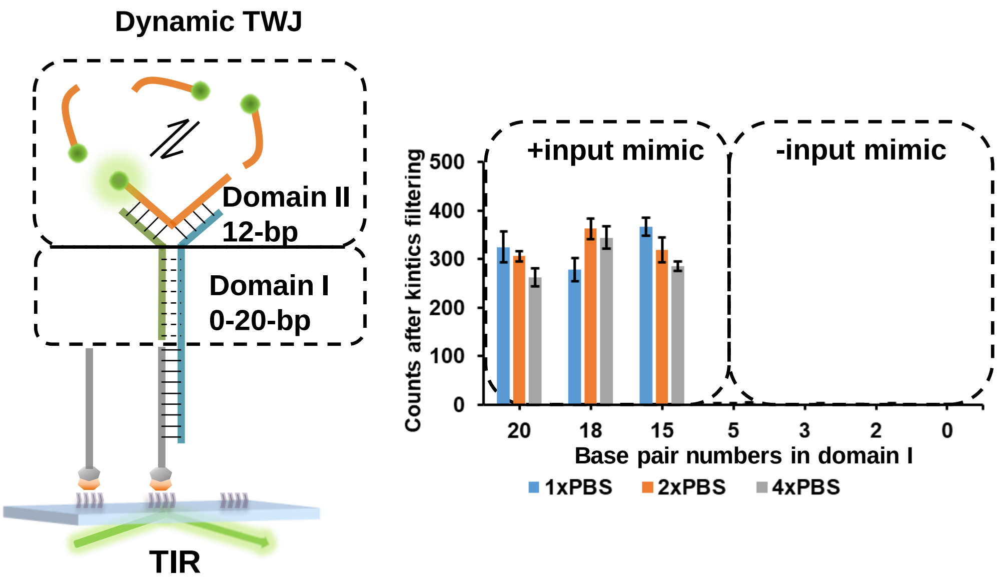

Figure S3 Single-molecule counts of the TWJs after kinetics filtering as a function of the length of the associate region (base pair numbers) in domain I. The base pair numbers in domain II is 12. The columns represent different salinity.

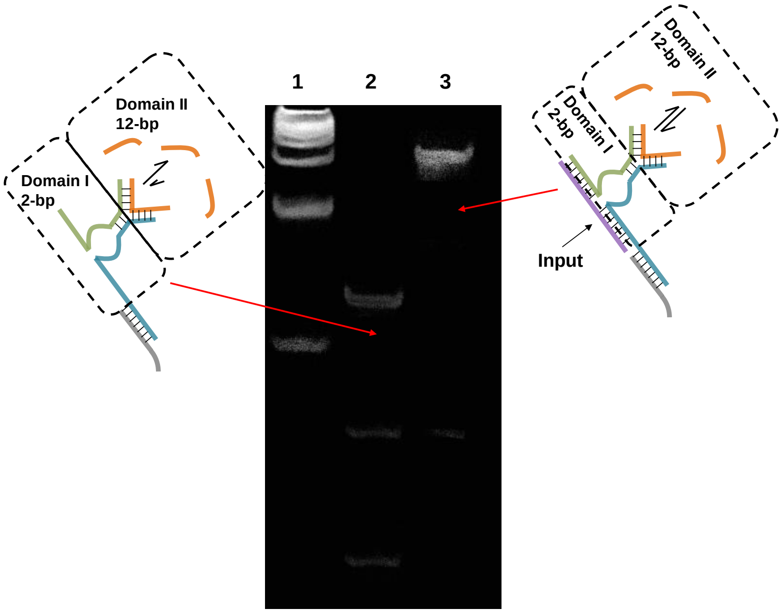

Figure S4 Native PAGE gel characterization of the DNA molecular switch (2-bp in domain I and 12-bp in domain II) for nucleic acid input. lane 1: 50-bp marker, lane 2: in the absence of the input, lane 3: in the presence of the input.

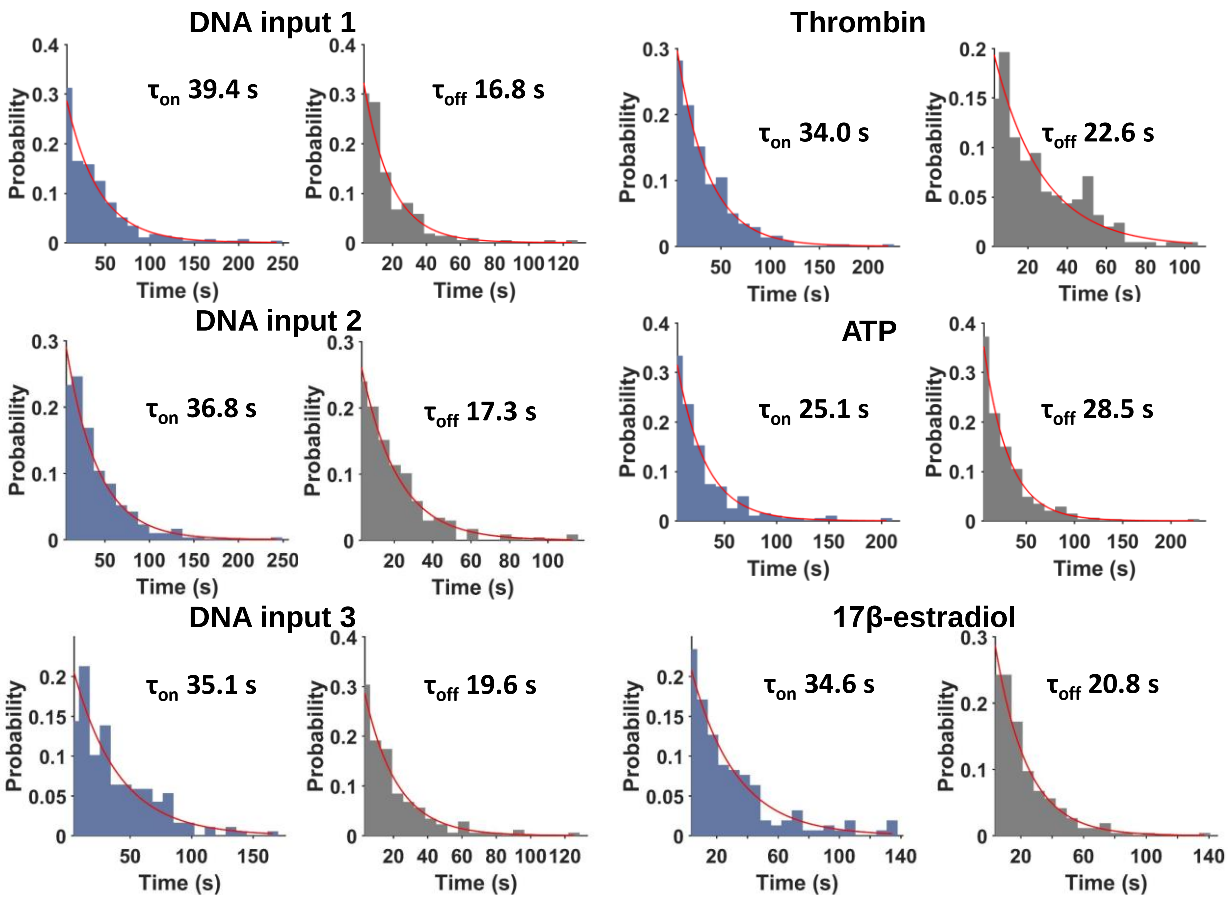

Figure S5 Dwell time distribution of the fluorescence-ON (τ_{on}) and -OFF (τ_{off}) states of the fluorescent probes in the molecular switches for different inputs. The sequences of DNA inputs (I1-I3) and the split aptamers of 17β-estradiol are shown in Table S1. The fluorescent probe, the capture probe, and the hybridization probe for DNA inputs are the same as those in Figure 2. The fluorescent probe and the capture probe for 17β-estradiol are the same as those in Figure 3. TWJ structures: 12-bp in domain II and 0-bp in domain I for 17β-estradiol, 12-bp in domain II and 2-bp in domain I for DNA inputs.

Reference

1. A. Johnson-Buck, X. Su, M. D. Giraldez, M. P. Zhao, M. Tewari and N. G. Walter, *Nat. Biotechnol.*, 2015, **33**, 730-732.
2. C. E. Aitken, R. A. Marshall and J. D. Puglisi, *Biophys. J.*, 2008, **94**, 1826-1835.
